# Effect of ice water pretreatment on the quality of Pacific White Shrimps (*Litopenaeus vannamei*)

**DOI:** 10.1002/fsn3.901

**Published:** 2018-12-04

**Authors:** Nan Xu, Wenzheng Shi, Xichang Wang, Zhihe Wang

**Affiliations:** ^1^ College of Food Science and Technology Shanghai Ocean University Shanghai China; ^2^ National R&D Branch Center for Freshwater Aquatic Products Processing Technology (Shanghai) Shanghai China

**Keywords:** ice water, Pacific White Shrimps, shell

## Abstract

Pacific White Shrimps (*Litopenaeus vannamei*) are an aquaculture species with global importance. For the purpose of this paper, the quality shelling process of Pacific White Shrimps, freshly harvested from farms and stored in a mixture ice water up to 24 hr, was investigated. Both the differences and correlations between the indexes such as peeling time, shrimp yield, chromatic aberration, texture, *K* value, TBA value, and microstructure were compared and analyzed. The optimal shell peeling time of Pacific White Shrimps was determined by ice water treatment for 8 hr. The shell peeling time was 1.77 min, the elasticity of the shrimp was 0.51 mm, the hardness was 2,124.58 g, *K* value was 1.33%, and TBA value was 0.004 mg/100 g. Both the smell and color of the shelled shrimp were normal. This study aims to endorse the mechanical shelling of Pacific White Shrimps.

## 1 INTRODUCTION

Pacific White Shrimps (*Litopenaeus vannamei*) are an aquaculture species native to the Pacific coast of Mexico. And commonly caught or farmed as an important food source. It is rich in protein, cellulose, mineral elements, vitamins, and a variety of amino acids that are beneficial to the human body (Zhang, Ma, Deng, Xie, and Qiu, 2015). Over the past 20 years, continuous advancement of shrimp farming technology in China and the widespread production of new aquaculture breeds have become more apparent. Following this trend, the shrimp production methods have gradually embraced the notion of aquaculture where these crustaceans are readily cultivated for the public market, rather than being captured solely from the nature habitat. Aquaculture production of shrimp has proportionately increased when viewed within the context of total shrimp production. Shrimp farming has developed rapidly, especially in the coastal regions of southern China, due to a suitable climate and abundant water resources and has become a pillar industry in the area (Chen & Kong‐Yue, 2012; Cheng & Zeng, 2011).

Since shrimp meat is one of China's most significant export products, the shelling process is a crucial step during processing. However, at present, shrimp are still shelled manually in China. Even though a handful of mechanical shrimp peelers exist, they have distinct disadvantages. The shrimp are delicate and are easily crushed in these machines. Moreover, both the efficiency and the output of processed shrimp are low when utilizing mechanical shrimp peelers. Various alternative methods are available for peeling shrimp, including low temperature shelling, ice salt shelling, blanching shelling, high pressure shelling, and flash freeze shelling (Liu, Liu, Wang, & Qin, 2014; Yang et al., 2010; Yi et al., 2012).

Currently, placing shrimps on ice and/or in brine (typically NaCl or NaCl with phosphates) solution for several days is the most common practices to facilitate the separation of the edible meat from the shell in the shrimp industry (Dang et al., 2018). Stern (1958) has reported that shrimp processed on ice can be peeled more easily than those freshly caught. Another study found by Taylor (1993) that manufacturers determined long ago that shrimps on ice for 3 or 4 days after catching greatly enhanced the ability of the peeling machine to remove the shell from the meat. To find a better way, Collins (1960) investigated the maturation of shrimp in frozen brine that simulated the refrigerated seawater and compared it with the ice method. Shrimps that have been treated in brine can be easily removed by hand with a machine and retained better color than the shrimps treated on ice. The ice treatment was simply layering the shrimp with flake ice in wooden bins for 40–48 hr, while brine treatment was soaking shrimps in a 3% and 6% sodium chloride solution at −1°C for 40 hr. The main advantages of shrimps treated in the brines were peeled satisfactorily by machine and easily by hand and retained better color than the shrimps treated on ice. Liu, Shen, Liu, and Qin (2013) and Liu et al. (2014) studied the effects of ice salt pretreatment and low temperature brine on the shelling of *Penaeus vannamei*. It was found that the low temperature brine and ice salt pretreatment not only reduced the difficulty of shell peeling, but also achieved the effect of low temperature preservation. However, the salt water concentration is higher, and the sample is easier to be frozen quickly, which led to the reduction of shell peeling efficiency and the serious loss of shrimp. Also, with the extension of soaking time, salt is easy to penetrate into the shrimp, affecting the quality of the shrimp.

Ice water pretreatment for shelling is to soak freshly caught shrimp in ice water overnight, followed by removing the shells. Not only it can solve the problem of cooling too fast caused by excessive salt concentration, but also it can reduce the effect of salt on the meat quality. Of course, the quality of the shrimp and the shelling time are closely related to how long it is soaked in the ice water. Extended soaking time eases the removal of the shrimp shells, but the overall quality of the shrimp is reduced.

The aim of this study was to evaluate the quality changes and shelling difficulty of Pacific White Shrimps (*Litopenaeus vannamei*), which were freshly harvested from farms and stored in ice water for up to 24 hr.

## 2 MATERIALS AND METHODS

### 2.1 Shrimp collection and preparation

Pacific White Shrimps were purchased from an aquatic products market in Shanghai, China, in mid‐January, 2018. It was harvested from local ponds and sent to the laboratory within half an hour while still alive. Dead killed and dying shrimp were discarded, while the remainder was evenly divided into seven groups of approximately 550 ± 5 g. The first group was used as a control group, and the shells were peeled and measured. Ice water was added to the remaining six groups. Samples were taken every 4 hr to determine shelling time and quality changes. The treated samples were stored at −80 ± 1°C for further use.

### 2.2 Shrimp shelling time

Samples that had been submerged in ice water for every 4 hr were removed and their weight recorded. Five people with experience in shelling shrimp were selected and provided with these samples for manual processing. Upon completion, both the weight and the time required for the shells to be removed were documented.

### 2.3 Sensory evaluation

The sensory analysis was conducted using whole Pacific White Shrimps for the shelling process. The five panelists were regular consumers of shrimp and had no allergies to shrimp, aged 20–30, three women and two men were trained according to standard ISO 8586‐2012, were selected to evaluate the color, the odor, the texture, and shelling ease of the shrimp. Each component received a score on a continuous scale of demerit points from 1 to 10, as changes occurred during storage (Table 1).

**Table 1:** Attributes assessed during the sensory evaluation of fresh Pacific White Shrimps

Parameter	Score		
	0–3	4–6	7–10
Color	Black becomes bigger	Body slightly red or black	Shiny body surface, normal color
Odor	Slight ammonia odor	Neutral odor	Inherent smell of shrimp
Texture	Less meat quality, softer muscle tissue	Better meat quality, denser muscle tissue	Good meat quality, dense muscle tissue
Shell meat separation	Clingy, difficulty	Fairly easy	Separate, easy

### 2.4 ATP‐related compounds and *K* value

ATP‐related compounds were extracted according to the procedure described by Liu, Jiang, Shen, Luo, and Gao (2015) with some modifications. 5 g of shrimp muscle was homogenized with 10 ml of cold perchloric acid (10%, v/v) by using an FM‐200 Homogenizer (Fluko Equipment Shanghai Co. Ltd, Shanghai, China) for 20 s and centrifuged at 13,800 g for 15 min at 4°C. The sediment was washed with 5 ml of cold perchloric acid (5%, v/v) and centrifuged under the same condition. This process was repeated twice. The combined supernatant was adjusted to pH 6.5 with 1 mol/L KOH solution and left to stand for 30 min. The supernatant was diluted to 50 ml with ultrapure water, through a 0.22 mm diameter filter and stored at −20°C for further analysis.

ATP‐related compounds (ATP, ADP, AMP, IMP, HxR, and Hx) were analyzed using high‐performance liquid chromatography (HPLC) (Shimadzu, LC‐10AT series, Kyoto, Japan) which was equipped with an SPD‐10A (V) detector and a COSMOSIL 5C18‐PAQ column (4.6ID × 250 mm). The mobile phase was 0.05 mol/L phosphate buffer at pH 6.5. The sample of 10 μl was injected at a flow rate of 1 ml/min, and the detection wavelength was set at 254 nm. *K* value was calculated using the following equation (Saito, 1959):

(1) $$K=\frac{\left(\text{HxR})+(\text{Hx}\right)}{\left(\text{ATP})+(\text{ADP})+(\text{AMP})+(\text{IMP})+(\text{HxR})+(\text{Hx}\right)}\times 100\%$$

where ATP, ADP, AMP, IMP, HxR, and Hx are content of adenosine triphosphate, adenosine diphosphate, adenosine monophosphate, inosine monophosphate, hypoxanthine riboside, and hypoxanthine, respectively.

### 2.5 Color measurements of the shrimp

The color of the shrimp was measured using an automatic chromatic aberration meter (DC‐P3, Shanghai Go On Chemical Co., Ltd., Shanghai, China) according to the method of Zhang et al. (2015). The color was determined from the second abdomen of the shrimps; the L* value (lightness: L* = 0 for black, L* = 100 for white), a* value (red/green: +a* = redness, −a* = greenness), and b* value (yellow/blue: +b* = yellowness, −b* = blueness) were recorded. Each group of samples was measured eight times, the results were recorded, and the average values were obtained. The instrument used a standard whiteboard for advanced correction. The total color differences (DE values) indicating the degree of chromatic aberration between the shrimp samples before and after storage were calculated using the following equation:

(2) $$\Delta E={\left[{\left(L^{*}-L_{0}^{*}\right)}^{2}+{\left(a^{*}-a_{0}^{*}\right)}^{2}+{\left(b^{*}-b_{0}^{*}\right)}^{2}\right]}^{1/2}$$

where L_0_*, a_0_*, and b_0_* denote the color values of the whiteboard.

### 2.6 Texture measurements

A texture profile analysis (TPA) of the shrimp muscle was performed by using a texture analyzer (TMS‐PRO, FTC, Sterling, VA, USA). The sample was placed on a platform, and a P/50 cylindrical Perspex probe (50 mm diameter) simulated the chewing process. The TPA was performed under the following conditions: constant test speed, 1.0 mm/s; sample deformation, 50%; hold time between cycles, 3 s; and trigger force, 0.05 N. The TPA parameters were calculated from the force‐time curves generated from each sample using the FTC‐PRO software. Each test was repeated eight times (Zhang et al., 2015).

### 2.7 Thiobarbituric acid (TBA) and pH

The samples were analyzed for thiobarbituric acid reactive substances (TBARS) using the modified method introduced by Faustman (Faustman, Specht, & Malkus, 1992). A 5 g sample was homogenized with 25 ml 20% of trichloroacetic acid (TCA) extraction solution and 20 ml of distilled water, and then centrifuged at 11,040 g for 10 min. A quantity of 5 ml supernatant was collected and mixed with 5 ml TBA (0.02 mol/L), and then heated in a water bath at 95°C for 20 min. The samples were cooled in cold flowing water and immediately loaded into a UV2450 ultraviolet spectrophotometer (Shimadzu, Japan) for measurement at 532 nm. Instead of the filtrate, distilled water was used as a blank sample. The specimen was measured in parallel, and the process was repeated three times to obtain an average value. The TBA value = A × 7.8 mg/100 g.

Refer to the method of Lan et al. (2014) and make minor modifications. Take shrimp 5 g, add 45 ml of distilled water, homogenize, stand for 30 min, centrifuge at 13,800 *g* for 10 min, filter, and take the supernatant to measure pH, parallel three times.

### 2.8 Basic nutrients

The moisture content, ashes, crude protein (Nx6.25), and lipids were measured in the raw shrimp according to AOAC (1997).

### 2.9 Scanning electron microscopy (SEM) analysis

The cross section of shrimp muscle was chemically prefixed with 2.5% (v/v) glutaraldehyde in 0.1 mol/L at pH 7.2 phosphate‐buffered saline (PBS) at 4°C and left to stand overnight. The samples were then postfixed with 1.0% (w/v) osmium tetroxide at room temperature for 1 hr. The specimens were washed three times in 50 mM of pH 7.2 PBS and then dehydrated for 15 min in a graded series of ethanol solution at 30%, 50%, 60%, 70%, 80%, 90%, 95%, and 100%, respectively. A critical‐point drier was employed to dehydrate the muscle using liquid CO_2_ (SFD, Thar Instruments, Inc., Pennsylvania, USA), followed by treatment in a gold‐coater (Eiko IB3, JEOL Ltd., Tokyo, Japan) for 5 min, and examined using an SEM (JSM‐6390LV, JEOL Ltd.) (Zhang, Fang, Hao, & Zhang, 2018).

### 2.10 Data processing

The results obtained from the various experiments conducted in this study were subjected to an analysis of variance (ANOVA). A simple correlation and regression analysis were used for the comparison of the parameters. The least‐square difference (LSD) method (Statsoft, 1994) was applied to identify any notable differences in means. The level of significance was defined at *p *<* *0.05, and the results were expressed as the mean ± standard deviation (*SD*). The data were analyzed statistically using IBM SPSS Statistics program for Mac (IBM SPSS Version 22.0, IBM Corporation, New York, USA). The figures were generated using Origin 8.5 (Origin Lab, Massachusetts, MA, USA).

## 3 RESULTS AND DISCUSSION

### 3.1 Determination of shrimp shelling time and shrimp yield

**Figure 1:**
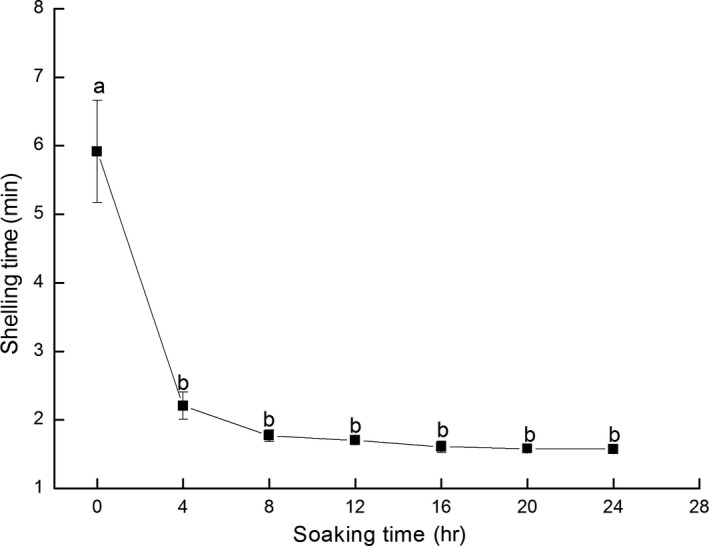
Changes in the shelling time of Pacific White Shrimps treated by ice water

The time that was required to remove the shrimp shells indicated the degree of difficulty of the shelling process. Thus, a shorter peeling time signified less difficulty in removing the shell from the meat. Figure 1 illustrates that the peeling time seemed to decline as the soaking time was extended. Fresh Pacific White Shrimps proved challenging to shell and it took shelled for 5.92 min. After 4 hr of immersion in ice water, the shelling time decreased significantly to 2.21 min. The shelling time ultimately dropped to 1.57 min after 24 hr treatment. The results indicated that a decrease in temperature during the processing of Pacific White Shrimps reduced the shelling problem substantially. No significant difference in the shelling time was evident after 4 hr treatment.

**Figure 2:**
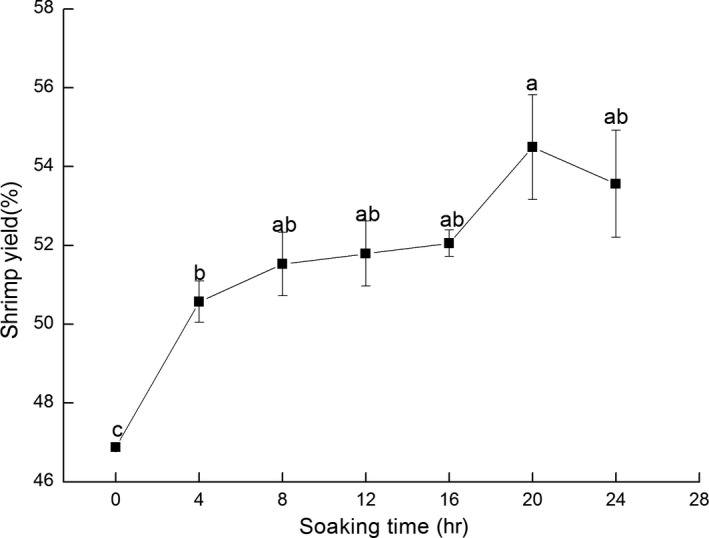
Shrimp yield change of Pacific White Shrimps treated by ice water

The shrimp yield reflects the output rate of shrimps after shelling (Yi et al., 2012). The control group was represented by 0 hr, and the shrimp yield was 46.87%. The shrimp yield increased considerably with prolonged time reaching 50.53% after 20 hr treatment. However, no significant difference was apparent in the sample group after an 8 hr treatment (Figure 2). This result indicated that the internal tissue cells of the shrimp body absorbed the external moisture during the process of ice water treatment, resulting in an increase in the total weight of the shrimp. The optimal treatment time was determined to be 8 hr and was calculated by combining the shell peeling time and shrimp yield.

### 3.2 Comparative sensory quality

According to the results of the sensory analysis, Pacific White Shrimps stored in slurry ice maintained a good quality (Table 2). With the extension of the ice water pretreatment time, the evaluation scores assigned to the texture, smell, and color indexes of Pacific White Shrimps exhibited a decreasing trend. A similar tendency is visible in the research of Liu (2014). The unpeeled Pacific White Shrimps with a pretreatment time of 0 hr displayed the highest score and the best quality. However, at 0 hr treatment, the shelling process proved to be exceptionally difficult and scored the lowest regarding the separation of the shell from the meat. A pretreatment time of between 4 and 8 hr promoted an easier peeling process, with 8 hr considered the optimal pretreatment time for Pacific White Shrimps.

**Table 2:** Sensory evaluations of fresh Pacific White Shrimps during the ice water storage period

	Pretreatment time						
	0	4	8	12	16	20	24
Texture	9.40 ± 0.55^a^	8.40 ± 0.55^b^	8.40 ± 0.55^b^	7.80 ± 0.45^bc^	7.20 ± 0.84^c^	6.25 ± 0.96^d^	6.20 ± 0.45^d^
Odor	8.80 ± 0.84^a^	8.20 ± 0.45^ab^	7.60 ± 0.55^bc^	7.20 ± 0.84^cd^	6.60 ± 0.55^de^	5.80 ± 0.84^ef^	5.00 ± 0.71^f^
Color	9.60 ± 0.55^a^	8.40 ± 0.55^b^	7.60 ± 0.55^c^	7.40 ± 0.55^c^	6.20 ± 0.45^d^	5.40 ± 0.55^e^	4.60 ± 0.55^f^
Shell meat separation	1.80 ± 0.84^c^	7.40 ± 0.55^a^	7.60 ± 0.55^a^	6.20 ± 0.45^b^	6.00 ± 0.80^b^	6.25 ± 0.96^b^	6.00 ± 0.82^b^

*Note*

Values are given as mean ± standard deviation (*SD*). Different letters in the same row indicate significant differences (*p* < 0.05).

### 3.3 Changes in ATP‐related compounds and effect on *K* value

AMP and IMP are degradation products of ATP and are two of the most important compounds that affect the flavor of the shrimp meat. These two nucleotides are responsible for the pleasant, fresh, sweet taste of shrimp meat. There are two ways of ATP degradation in shrimp after death. One is ATP → ADP → AMP → IMP → HxR → Hx, and the other is ATP → ADP → AMP → AdR → HxR →Hx. Table 3 shows the changes in ATP‐related compounds during the ice water storage period. The content of the taste nucleotide AMP in the sample treated for 0 hr was the largest, about 203.98 mg/100 g. Furthermore, the IMP content was 23.73 mg/100 g, and a small amount of the bitter nucleotides Hx and HxR were detected. With an increase in soaking time of Pacific White Shrimps sample in ice water, the ATP, ADP, and AMP levels generally decreased, while the content of IMP increased. This result indicated ATP was rapidly degraded to AMP through ADP by enzyme action during the ice water treatment of the shrimp. AMP was broken down into IMP by AMP deaminase. IMP is an intense flavor substance found in shrimp and when it is present in high concentrations, it is associated with the appetizing quality of shrimp meat. After 24 hr, the IMP content reached 125.62 mg/100 g, while the HxR content of fresh Pacific White Shrimps was 1.95 mg/100 g and displayed elevated levels of 2.82 mg/100 g after 12 hr, followed by decrease to 2.25 mg/100 g after 24 hr. The increase of the HxR value was due to the dephosphorylation of IMP under the action of 5′‐nucleotidase. The reduction in HxR content might be due to the metabolites produced by the proliferation of the shrimp during the storage period and the interaction of endogenous enzymes to promote HxR degradation (Qiu et al., 2015).

**Table 3:** Changes in ATP‐related compounds of fresh Pacific White Shrimps during the ice water storage period

	Contents (mg/100 g)						
	0 hr	4 hr	8 hr	12 hr	16 hr	20 hr	24 hr
ATP	6.16 ± 1.01^bc^	5.06 ± 0.92^cd^	3.80 ± 0.61^de^	6.82 ± 0.59^ab^	8.13 ± 0.05^a^	3.47 ± 0.02^e^	1.83 ± 0.09^f^
ADP	19.92 ± 0.31^a^	18.12 ± 0.37^ab^	17.19 ± 0.65^ab^	18.77 ± 2.40^ab^	15.86 ± 2.38^b^	16.53 ± 0.07^b^	16.16 ± 0.59^b^
AMP	203.98 ± 6.55^a^	193.65 ± 1.99^a^	164.72 ± 3.73^b^	157 ± 9.49^bc^	136.85 ± 12.28^cd^	129.28 ± 18.70^d^	131.31 ± 6.02^d^
IMP	23.73 ± 6.02^e^	14.87 ± 0.00^e^	54.94 ± 8.72^d^	88.21 ± 2.44^c^	108.10 ± 1.55^b^	114.08 ± 0.81^b^	125.62 ± 2.68^a^
HXR	1.95 ± 0.18^d^	2.17 ± 0.29^cd^	2.65 ± 0.25^abc^	2.82 ± 0.05^a^	2.50 ± 0.2^abc^	2.74 ± 0.06^ab^	2.25 ± 0.26^bcd^
HX	0.71 ± 0.01^bc^	0.84 ± 0.01^ab^	0.60 ± 0.05^c^	0.89 ± 0.08^a^	0.66 ± 0.05^c^	0.91 ± 0.12^a^	0.90 ± 0.00^a^
Total	256.45 ± 2.06^abc^	234.71 ± 3.00^c^	243.92 ± 6.46^bc^	274.52 ± 8.89^a^	272.09 ± 16.42^a^	267.01 ± 17.66^ab^	278.07 ± 9.46^a^

*Note*

Values are given as mean ± standard deviation (*SD*). Different letters in the same row indicate significant differences (*p* < 0.05).

**Figure 3:**
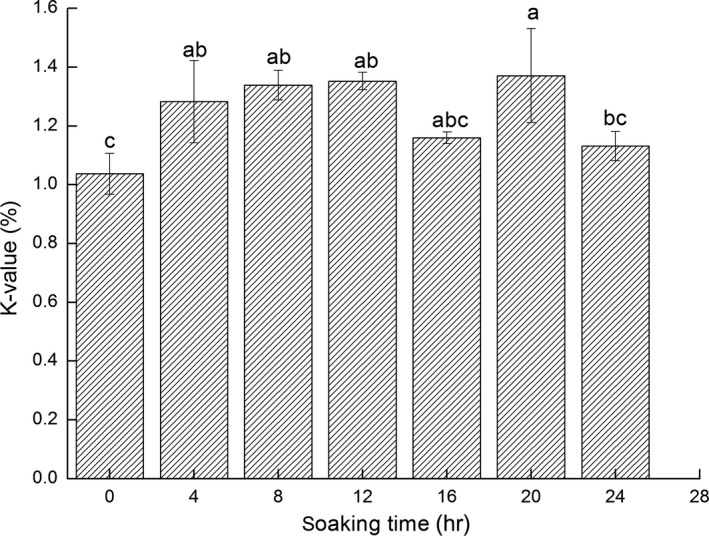
Changes in *K* value of fresh Pacific White Shrimps during the ice water storage period

The *K* value is a product produced by disintegration of nucleotides and is used as an index to determine changes in the freshness of fish. The fish is fresher when a smaller *K* value is present, while a more substantial *K* value indicates deterioration in freshness. The *K* value of fresh fish is below 20%, 20%~40% reflects secondary freshness, and 60%~80% indicates the initial stages of the fish turning bad. Figure 3 demonstrates an initial increase in the *K* value followed by a decline. This result was consistent with the overall pattern of changes in HxR and Hx. The *K* value was below 2%, indicating that the tested Pacific White Shrimps retained first‐grade freshness.

### 3.4 Changes in color

L*, also known as lightness, is an amalgamation of achromatic colors, which appear as black, gray, and white ranging from dark to bright.Generally, the color became brighter the larger the L* value is (Zou et al., 2010). It is seen from the data in Table 4 that L* increased in conjunction with the elevation of ice water treatment time. The L* value of the soaked sample was significantly higher than that of the control group (0 hr) (*p *<* *0.05) and reached the maximum value of 47.21 after 20 hr. The increase in the L* of the shrimp color was mainly due to the formation of ice crystals in the holding water containing the shrimp meat that resulted in a decrease in the level of ice water. Consequently, the ice water level was reduced, exposing more shrimp on the surface and increasing the light reflection effect (Pearson & Dutson, 1985).

**Table 4:** Changes in color of fresh Pacific White Shrimps during the ice water storage period

	Pretreatment time (hr)						
	0	4	8	12	16	20	24
L*	37.94 ± 2.82^c^	40.47 ± 2.81^bc^	40.83 ± 4.2^bc^	41.64 ± 2.84^b^	42.02 ± 1.81^b^	47.21 ± 4.43^a^	41.75 ± 2.36^b^
a*	−0.87 ± 0.48^a^	−0.92 ± 0.68^a^	−1.12 ± 0.72^a^	−1.16 ± 0.28^a^	−0.79 ± 0.09^a^	−1.40 ± 0.27^a^	−0.79 ± 0.23^a^
b*	−1.68 ± 0.83^ab^	−1.73 ± 0.79^ab^	−0.96 ± 0.81^a^	−2.29 ± 0.53^b^	−2.16 ± 1.3^b^	−2.18 ± 0.68^b^	−2.13 ± 0.78^b^

*Note*

Values are given as mean ± standard deviation (*SD*). Different letters in the same row indicate significant differences (*p* < 0.05).

The symbol a* represents the value of redness, and b* is the yellowing value. Due to an abundance of polyphenol oxidase in shrimp, it reacted effortlessly with DOPA substances to produce melanin (Bartolo & Birk, 1998). According to the data in Table 4, the values of a* and b* in the South American white prawn did not exhibit significant change in the short term (*p *>* *0.05). The observation seemed to agree with the color data reported by Okpala (2015) regarding the pink shrimp (*Parapenaeus longirostris*) that were processed onboard the shrimp under iced storage conditions.

### 3.5 Changes in texture

Following the demise of marine creatures such as shrimp, a decline in freshness and decay occur due to microorganism activity. This decomposition process leads to changes in the structure of muscle tissue and texture properties, causing the softening and elasticity of aquatic products. The quality of aquatic products is determined by essential attributes relating to its textural properties including hardness, springiness, cohesiveness, gumminess, and chewiness, and recovery hardness and elasticity are the most likely qualities to undergo changes in aquatic products (Botta, 1994). The changes in the texture qualities of Pacific White Shrimps during treatment in an ice water mixture are shown in Table 5.

**Table 5:** Changes in texture of fresh Pacific White Shrimps during the ice water storage period

	Pretreatment time (hr)						
	0	4	8	12	16	20	24
Hardness	2,011.70 ± 205.78^b^	2,022.38 ± 330.21^b^	2,124.58 ± 172.19^ab^	2,006.95 ± 192.62^b^	2,291.50 ± 607.64^ab^	2,349.77 ± 132.71^ab^	2,562.43 ± 193.90^a^
Springiness	0.72 ± 0.06^a^	0.60 ± 0.07^b^	0.51 ± 0.07^c^	0.50 ± 0.04^c^	0.50 ± 0.03^c^	0.51 ± 0.02^c^	0.49 ± 0.03^c^
Cohesiveness	0.47 ± 0.04^a^	0.42 ± 0.04^b^	0.40 ± 0.03^b^	0.40 ± 0.02^b^	0.37 ± 0.03^b^	0.38 ± 0.02^b^	0.39 ± 0.03^b^
Gumminess	857.85 ± 135.04^a^	850.89 ± 114.87^a^	877.07 ± 129.06^a^	884.49 ± 66.05^a^	892.77 ± 135.8^a^	999.93 ± 53.69^a^	991.67 ± 137.24^a^
Chewiness	454.43 ± 145.63^a^	465.10 ± 63^a^	477.49 ± 20^a^	447.79 ± 68.15^a^	462.51 ± 76.73^a^	473.4 ± 89.59^a^	476.03 ± 47.18^a^
Resilience	0.29 ± 0.01^a^	0.25 ± 0.02^b^	0.24 ± 0.02^b^	0.24 ± 0.02^b^	0.25 ± 0.02^b^	0.24 ± 0.01^b^	0.25 ± 0.02^b^

*Note*

Values are given as mean ± standard deviation (*SD*). Different letters in the same row indicate significant differences (*p* < 0.05).

**Figure 4:**
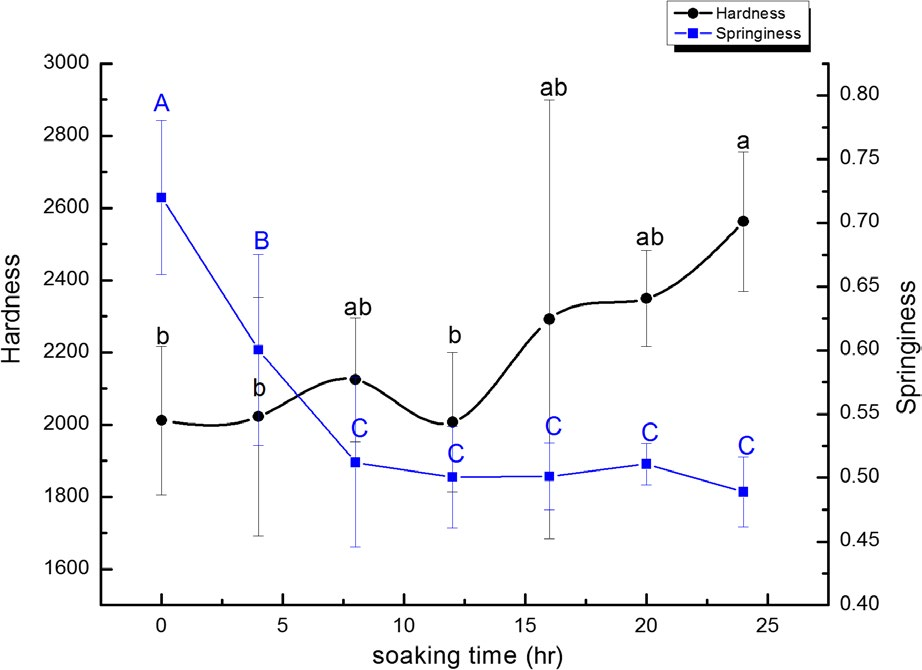
Changes in hardness and springiness of fresh Pacific White Shrimps during the ice water storage period

Hardness refers to achieving peak force at the first compression of force‐time in a typical texture analysis profile (Chen, 2009). Hardness is the most critical textural attribute in meat or seafood (Szczesniak, 2002). Table 5 and Figure 4 clearly show that with the increase of the ice water immersion time, the hardness of the Pacific White Shrimps showed an upward trend, but the range of variation was minimal. There was a significant difference (*p *<* *0.05) in the hardness of the shrimp samples following immersion in ice water for 0 hr (2,011.70 g), 4 hr (2,022.38 g), and 24 hr (2,562.43 g), respectively. Following the death of aquatic products, the body is tightness and the hardness increases. Due to changes in the structure of collagen molecules, myofibril becomes fragile, due to the activity of enzymes and microorganisms, the muscle cytoskeleton protein, and the degradation of the extracellular matrix structure. Consequently, the structure between the myofibril is loosed, resulting in muscle texture softening, decreased elasticity, and quality deterioration (Magnussen, Haugland, Akt, Johansen, & Nordtvedt, 2008; Steen, Claeys, Uytterhaegen, De, & Demeyer, 1997; Taylor, Fjaera, & Skjervold, 2010). Therefore, during this process, the hardness value initially increases followed by a declining trend. However, the longevity of the rigidity period in marine products depends on a multitude of factors including the physiological state, endogenous enzyme activity, individual size, lethal mode, and storage methods. It is possible that the shrimp samples continued to display signs of stiffness after 24 hr due to the potential effect that the frigid temperature of the water had on the enzyme activity and the degradation of the protein.

Furthermore, elasticity is another critical evaluation index to determine the quality of texture. Elasticity is the ratio of height or volume of a denatured sample to its predeformation height after removal of the deforming forces. The flexibility of the sample continued to decline throughout the treatment period (Table 5 and Figure 4). The sample exhibited the ideal elasticity of 0.72 mm at 0 hr, followed by the 4 hr sample with an elasticity of 0.62 mm. It was evident that the elasticity value fluctuations remained within a specific range after 4 hr. This result indicated that the low temperature could both lead to the decrease of elasticity and inhibit this elasticity decline to a certain extent. The hardness and elasticity measurements were consistent with those of Ando, Nakamura, Harada, and Yamane (2004) and Yuan, Zhang, Tang, and Sun (2016) and Wang, Zeng, Dong, Liu, and Zhao (2011).

Cohesiveness represents the cohesive force inside the sample. Resilience refers to the degree of recovery of the deformed sample at the same speed and pressure as the deformation. Chewiness refers to the energy required for food to be chewed until it reaches a stable state suitable to swallow. From Table 5, it is clear that the cohesion and resilience generally show a downward trend during the treatment process, and there is a significant difference (*p *<* *0.05) between all samples after 4 hr treatment and the control group. Furthermore, adhesiveness and chewiness did not change significantly during the treatment (*p *>* *0.05).

### 3.6 Changes in pH and TBA

**Figure 5:**
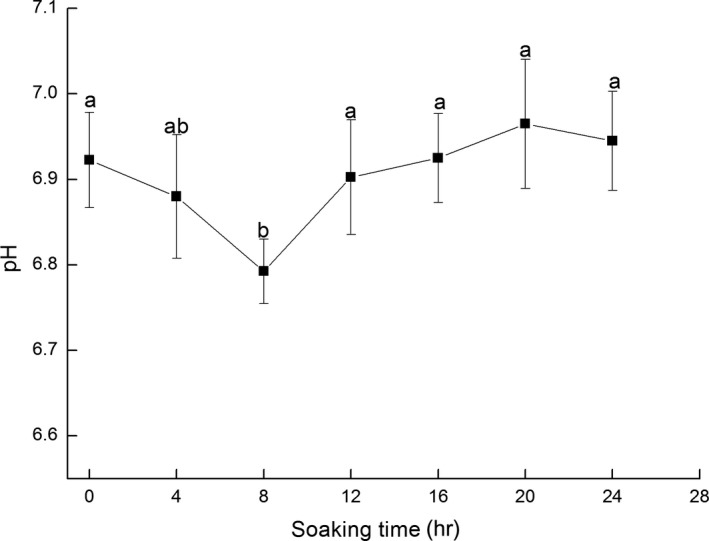
Changes in pH value of fresh Pacific White Shrimps during the ice water storage period

Measuring changes in the pH value of shrimp muscle can be used as one of the reference standards for judging its freshness (Kural, Shearer, Kingsley, & Chen, 2008). Generally, after an aquatic animal stops breathing, the glycogen in its body is degraded to produce acid substances such as lactic acid, which causes the pH in the muscle to decrease. The degree of decline is related to the amount of glycogen in the muscle. However, an increase in muscle pH became apparent when the proteins, amino acids, and other nitrogen‐containing substances in the shrimp were transformed into basic materials such as ammonia, trimethylamine, indole, and histamine by the enzyme and microorganism activity during the decomposition process (Huss, 1995). Therefore, the variation trend of muscle pH value was generally V‐shaped meaning that the pH was change in shrimp after treatment with ice water: first decreased and then increased (Figure 5). The initial pH value in the muscle of Pacific White Shrimps was about 6.92. The time was extended, and the pH value declined to its lowest level after 8 hr, followed by a gradual increase. The pH value of the fresh shrimp was typically above 6 (Montero, Ávalos, & Pérez‐Mateos, 2001), indicating that ice water pretreatment had no adverse effect on the freshness of Pacific White Shrimps. However, pH levels between 6.5 and 7.5 provide ideal conditions for bacterial growth and continued ice water treatment promoted the decay of the Pacific White Shrimps.

**Figure 6:**
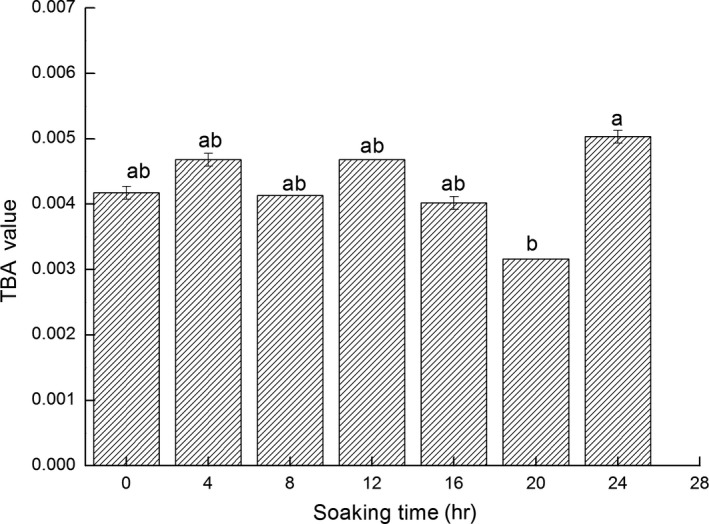
Changes in TBA value of fresh Pacific White Shrimps during the ice water storage period

Fat is easily corroded and broken down into malondialdehyde (MDA) that reacts effortlessly with TBA to create a stable red compound (Yanar & Fenercioǧlu, 2014). Therefore, the higher the fat oxidation was, the more substantial the deterioration of the Pacific White Shrimps (Qiang, Zhang, Lu‐Kai, & Wang, 2014). This result indicated that the degree of fat oxidation in seafood could be reflected by changes in the TBA values. It is clear from Figure 6 that the TBA values were small, and no significant differences in the sample group with an increase in time (*p *<* *0.05). This indicated that the Pacific White Shrimps that was subjected to ice water treatment still exhibited a fat oxidation reaction. However, due to the low‐fat content in the shrimp meat, both the speed of the fat oxidation and the TBA value occurred gradually.

### 3.7 Basic nutrients

Generally, a shrimp consists of a head, a shell, the meat/muscle, and a tail. The meat is the main part, accounting for 48% of the total shrimp. The chemical analysis of fresh Pacific White Shrimps is presented in Table 6. It can be seen that the protein content in the muscle of Pacific White Shrimps is up to 17.01%, and converting into dry weight as high as 81.43, which is slightly lower than that of Mediterranean red shrimp (87.5) and black tiger shrimp (87.69), higher than Amazon shrimp (67.55), Macrobrachium rosenbergii (76.66), and Procambarus clarkii (76.19) (Bono et al., 2012; Feng, Han, Wang, Fu, & Yin, 2011; Martins et al., 2017; Sriket, Benjakul, Visessanguan, & Kijroongrojana, 2007). The crude fat content of Pacific White Shrimps was only 0.4% making it a typical high‐protein, low‐fat health food.

**Table 6:** Chemical analysis of fresh Pacific White Shrimps

Moisture (g/100 g)	Proteins (g/100 g)	Lipids (g/100 g)	Ashes (g/100 g)
79.11 ± 2.16	17.01 ± 1.23	0.40 ± 0.03	1.39 ± 0.11

### 3.8 Muscle tissue structure

**Figure 7:**
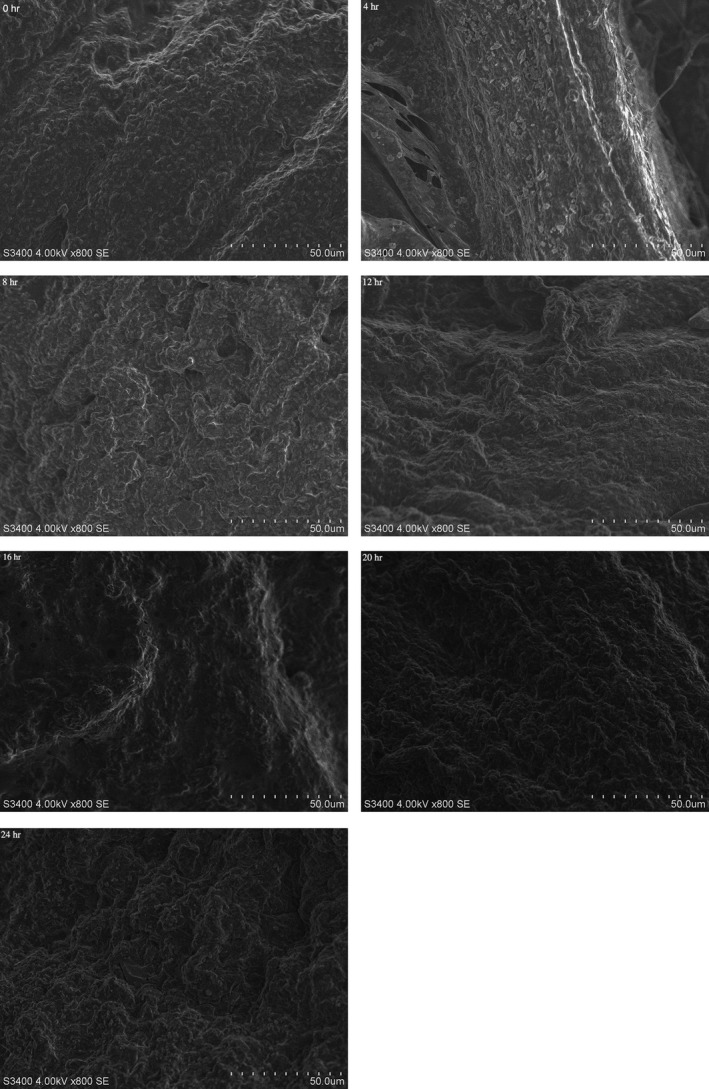
Muscle tissue structural change of Pacific White Shrimps during the ice water storage period

Under the low temperature conditions, the growth of ice crystals and the changes in muscle fibers prompted modifications to occur in the shrimp muscle tissue structure. The muscle bundles in the fresh specimen that underwent 0 hr of processing (Figure 7) were tightly connected to each other with small spaces evident between them indicating a well‐organized structure. Similarly, samples that were processed for 4 hr (Figure 7) displayed relatively clear muscle fiber texture that was compact with small space between. As the ice water treatment time was prolonged, the muscle fibers appeared to be broken and squashed. The sarcomeric space gradually increased, and the muscle fiber structure became dense and loose even though the difference was not apparent in the sample group that was processed for 4 hr. The reason for this might be attributed to the muscle tissue in the shrimp, especially the connective tissue, was degraded, which was responsible for the eventual increase in the sarcomere gap. However, the low temperature also inhibits the degradation of the connective tissue to some extent.

### 3.9 Correlation analysis

Table 7 indicates that the elasticity index negatively correlated with the brightness (L*) and the content of IMP (*p *<* *0.01), and positively correlated with the content of AMP (*p *<* *0.05). L* was negatively correlated with a*, the TBA value, and the content of AMP (*p *<* *0.05), and positively correlated with the content of IMP (*p *<* *0.05). Regarding nucleotide content, ADP was positively correlated with AMP (*p *<* *0.01), ADP was negatively correlated with IMP (*p *<* *0.05), and AMP was negatively correlated with IMP (*p *<* *0.01). The results obtained from the correlation analysis suggested that the texture, chromatic aberration, and nucleotide content were closely associated during the ice water treatment process. The elasticity index shows a high positive correlations with the AMP content and negatively correlated with the brightness L* and IMP content. This result indicated that when the elasticity and the AMP content increased, both the brightness L* value and IMP content decreased, and vice versa. During the analysis of the texture, chromatic aberration, and nucleotide content, it was revealed that the elasticity index showed a downward trend with the increase of treatment time, and the brightness L* value and IMP content were elevated, while the AMP content was significantly reduced. This was consistent with the results obtained from the correlation analysis was similar to the experimental results of Liu et al. (2014) and Zhang et al. (2015).

**Table 7:** Correlation analysis between indicators

	Hardness	Springiness	*L* *	*a* *	*b* *	pH	TBA	ATP	ADP	AMP	IMP	HxR	Hx
Hardness	1	−0.422	0.257	−0.134	−0.349	0.310	0.065	−0.678	−0.437	0.004	−0.307	0.404	0.033
Springiness		1	−0.447**	−0.017	0.288	−0.135	−0.148	0.126	0.485	0.814*	−0.885**	−0.434	−0.643
*L* *			1	−0.336*	0.094	0.315	−0.569*	−0.071	−0.409	−0.643*	0.621*	0.393	0.519
*a* *				1	−0.117	−0.352	−0.112	−0.217	−0.005	0.042	−0.010	−0.046	−0.213
*b* *					1	−0.020	−0.099	−0.324	0.131	0.349	−0.403	−0.121	−0.492
pH						1	0.135	0.128	−0.049	−0.240	0.317	0.517	−0.192
TBA							1	−0.053	0.398	0.224	−0.128	0.260	−0.226
ATP								1	0.242	0.260	−0.220	−0.400	0.053
ADP									1	0.762**	−0.610*	0.090	−0.193
AMP										1	−0.945**	−0.276	−0.521
IMP											1	0.312	0.490
HxR												1	0.119
Hx													1

*Notes*

The numbers in the table indicate the correlation coefficient. The closer the correlation coefficient is to 1 or −1, the stronger the correlation coefficient is, the closer the correlation coefficient is to 0, and the weaker the correlation coefficient is.

*Denotes the significant difference, and **Denotes the extremely significant difference.

## 4 CONCLUSION

The changes in shelling time, shrimp yield, sensory indexes, and physical and chemical indicators (texture, chromatic aberration, TBA value, *K* value, and microstructure) of shrimp were determined, and correlation analysis was carried out for each index. The optimum process conditions were determined to be ice water treatment for 8 hr. The results showed that proper ice water treatment can significantly reduce the difficulty of the shelling process of Pacific White Shrimps while maintaining the quality of the product. Therefore, controlling the treatment time can effectively improve the efficiency of shell removal, and proves to be a feasible pretreatment method. In addition to the low efficiency of manual shell peeling, easy pollution, and food safety, soaking time is also a factor limiting shrimp yield. Other shrimp peeling technologies (ultra‐high pressure, ultrasonic, microwave, enzymatic method, etc.) are research hotspots, too. And some achievements have been made. Under the premise of ensuring product quality, the physical method combined with enzymatic method can be a research direction, which can shorten the soaking time. The results of this study could provide excellent theoretical guidance in the processing procedure of shrimp.

## CONFLICT OF INTEREST

The authors declare that they do not have any conflict of interest.

## ETHICAL STATEMENT

This study does not involve any human testing.
